# Structure Prediction and Characterization of Thermostable Aldehyde Dehydrogenase from Newly Isolated *Anoxybacillus geothermalis* Strain D9

**DOI:** 10.3390/microorganisms10071444

**Published:** 2022-07-18

**Authors:** Nur Ezzati Rosli, Mohd Shukuri Mohamad Ali, Nor Hafizah Ahmad Kamarudin, Malihe Masomian, Wahhida Latip, Shazleen Saadon, Raja Noor Zaliha Raja Abd Rahman

**Affiliations:** 1 Enzyme and Microbial Technology Research Centre, Faculty of Biotechnology and Biomolecular Sciences, Universiti Putra Malaysia (UPM), Serdang 43400, Malaysia; ezzatirosli14@gmail.com (N.E.R.); mshukuri@upm.edu.my (M.S.M.A.); hafizah_kamar@upm.edu.my (N.H.A.K.); malihe.m@upm.edu.my (M.M.); wahhidalatip@gmail.com (W.L.); shazleen.saadon@petronas.com (S.S.); 2 Department of Microbiology, Faculty of Biotechnology and Biomolecular Science, Universiti Putra Malaysia (UPM), Serdang 43400, Malaysia; 3 Department of Biochemistry, Faculty of Biotechnology and Biomolecular Science, Universiti Putra Malaysia (UPM), Serdang 43400, Malaysia; 4 Centre of Foundation Studies for Agricultural Sciences, Universiti Putra Malaysia (UPM), Serdang 43400, Malaysia; 5 Department of Hydrocarbon Recovery Technology, PETRONAS Research Sdn Bhd, Lot 3288 & 3299, Off Jalan Ayer Hitam, Kawasan Institusi Bangi, Bandar Baru Bangi 43000, Malaysia

**Keywords:** aldehyde dehydrogenase, thermostable enzyme, biochemical characterization, biophysical characterization, thermophile

## Abstract

In nature, aldehyde dehydrogenase (ALDH) is widely distributed and mainly involved in the oxidation of aldehydes. Thermostability is one of the key features for industrial enzymes. The ability of enzymes to withstand a high operating temperature offers many advantages, including enhancing productivity in industries. This study was conducted to understand the structural and biochemical features of ALDH from thermophilic bacterium, *Anoxybacillus geothermalis* strain D9. The 3D structure of *A. geothermalis* ALDH was predicted by YASARA software and composed of 24.3% β-sheet located at the center core region. The gene, which encodes 504 amino acids with a molecular weight of ~56 kDa, was cloned into pET51b(+) and expressed in *E.coli* Transetta (DE3). The purified *A. geothermalis* ALDH showed remarkable thermostability with optimum temperature at 60 °C and stable at 70 °C for 1 h. The melting point of the *A. geothermalis* ALDH is at 65.9 °C. Metal ions such as Fe^3+^ ions inhibited the enzyme activity, while Li^+^ and Mg^2+^ enhanced by 38.83% and 105.83%, respectively. Additionally, this enzyme showed tolerance to most non-polar organic solvents tested (xylene, n-dedocane, n-tetradecane, n-hexadecane) in a concentration of 25% *v*/*v*. These findings have generally improved the understanding of thermostable *A. geothermalis* ALDH so it can be widely used in the industry.

## 1. Introduction

High protein thermostability is critical for organisms that live in high-temperature habitats, as well as biotechnological applications that utilize enzymes as biocatalysts under often-aggressive reaction conditions [1]. Because thermostable enzymes are very selective, they have a lot of potential for industrial application. The application of such enzymes in maximizing processes in food and paper industries, detergents, medicines, the removal of toxic wastes, and oil drilling is being investigated intensively [2]. The thermostable enzymes can be manufactured using either optimized microbe fermentation or recombinant DNA technologies to clone fast-growing mesophiles [2]. 

The thermostability of the enzymes is related to the structure of the enzymes. Increased protein thermostability has been ascribed to a number of characteristics, including stronger hydrogen bonding, ion pair and salt bridge networks, greater hydrophobic packing, reduced loops, and more secondary structure content, all of which favor increased folded state structural rigidity [3]. Kevin et al. (2018) reported that the quaternary structure of *Thermus thermophilus* aldehyde dehydrogenase (ALDHTt) is stabilized by an evolutionary distinct C-terminal arm extension [4]. The structure has non-canonical dimeric and tetrameric characteristics as well as a previously unknown C-terminal arm extension that forms unique contacts with the N-terminus in the quaternary structure. This structure is crucial to understand how metabolic regulation has developed, as well as the relationship to early enzyme regulatory adaptations. [4].

Aldehyde dehydrogenase (ALDH) is a polymorphic enzyme that converts aldehydes to carboxylic acids, both endogenously and exogenously. As a coenzyme, NAD^+^ or NADP^+^ is used to carry out the oxidation process [5]. This evolutionarily conserved enzyme can be found in three different life domains, namely: Eukarya, Bacteria, and Archaea [6]. More than 160 ALDH cDNAs or genes have been isolated and sequenced from various sources (e.g., bacteria, yeast, fungi, plants, and animals) [7,8]. ALDH is involved in a variety of metabolic pathways in the Eukarya, including detoxification, stress protection, and fatty aldehyde metabolism. ALDH in bacteria is also involved in a variety of biodegradation or biotransformation routes, including amino acid, myoinositol, catabolism, and benzoate oxidation [9,10]. 

Depending on their isolation sources, ALDH have different biochemical characteristics. Enzymes can be characterized based on temperature, pH, metal ions, substrates, organic solvents, and other characteristics. Knowledge on the properties of an enzyme may allow it to easily be applied in industry. ALDH has been applied in various industrial applications, mainly in the removal of toxic metabolites [1,9]. 

A newly isolated bacterium from seawater has been identified as *Anoxybacillus geothermalis* strain D9. Phylogenetic analysis showed 100% confidence that D9 was closely related to *Anoxybacillus rupiensis* R270. The missing gap deletion procedure was used to generate the phylogenetic connection using a partial 16S rRNA nucleotide sequence. The BLAST result for a complete 16S rRNA nucleotide sequence of D9 identified *Anoxybacillus geothermalis* ATCC BAA-2555 with 99.8% identity. *A. geothermalis* D9 utilizes crude oil, sucrose, glucose, and lactose as sole carbon sources, and can hydrolyze lipid, starch, and casein in 24 h incubation. This thermophilic bacterium is a gram-positive rod and endospore-forming bacteria [11]. 

This bacterium was found to produce ALDH. This paper reports on the structure prediction of the *A. geothermalis* ALDH, cloning, and expression of the thermostable ALDH from *Anoxybacillus geothermalis* strain D9. The detailed biochemical and biophysical properties of the enzyme were investigated. 

## 2. Materials and Methods

### 2.1. Bacterial Strains and Culture Conditions

The *Anoxybacillus geothermalis* strain D9 and *E. coli Transetta* (DE3) were obtained from the Enzyme and Microbial Technology (EMTech) Research Center, Faculty of Biotechnology and Biomolecular Sciences, Universiti Putra Malaysia. The bacteria were grown in Luria-Bertani (LB) medium at 60 °C and 37 °C, respectively, with 200 rpm shaking. An appropriate amount of chloramphenicol was added to the *E. coli Transetta* (DE3) culture medium.

### 2.2. Nucleotide Sequence Accession Number

The sequence of the *A. geothermalis* ALDH gene has been deposited in the GenBank database under accession number MT584808.

### 2.3. Structure Prediction and Validation of A. geothermalis ALDH 

Using templates from the Protein Data Bank (PDB) that are highly comparable to *A. geothermalis* ALDH, homology modelling was performed to predict its 3D structure using YASARA. YASARA provides automated methods for protein structure prediction. Figures were generated using the Chimera visual system (www.cgl.ucsf.edu/chimera, accessed on 14 March 2021), while online applications such as Ramachandran Plot (http://www-cryst.bioc.cam.ac.uk/, accessed on 16 March 2021), Errat (https://saves.mbi.ucla.edu/, accessed on 16 March 2021), and VERIFY 3D (https://saves.mbi.ucla.edu/, accessed on 16 March 2021) were used to validate protein structure.

### 2.4. Cloning and Transformation of A geothemalis ALDH Gene

The *A. geothermalis* ALDH gene was identified by QC and de novo assembly method. Paired-end Illumina sequences were first removed by sequence adaptors and reads with low quality scores using bbduk of the BBTools Packages. QC reads were assembled de novo using SPAdes. The genome (scaffolds > 1000 bp) was subsequently annotated using the RAST pipeline [12,13]. The *A. geothermalis* ALDH was filtered by using the Translate tool, which is ExPASy server (http://www.expasy.org/tools/dna.html, accessed on 20 February 2021). 

Genomic DNA from *A. geothermalis* strain D9 was extracted by DNeasy Blood and Tissue Kits (QIAGEN, Hilden, German) according to its manufacturer’s instruction. The *A. geothemalis* ALDH was amplified by polymerase chain reaction (PCR) using primers 5′-CATGGTACCAATGATTTATAACCGGC-3′ (*Kpn*1 restriction site is underlined) and 5′-TATGAGCTCTGAAAAACCCTTGCG-3′ (*Sac*1 restriction site is underlined). The PCR product and pET-51b(+) plasmid were double digested with *Kpn*1 and *Sac*1 and the resulting fragments were ligated using T4 DNA ligase. The presence of the insert was verified via Sanger sequencing using T7 promoter and T7 terminator primers. The constructed recombinant plasmid was transformed into *E. coli Transetta* (DE3) competent cell.

### 2.5. Expression and Purification of A geothemalis ALDH

The *E. coli Transetta* (DE3) carrying pET51b/*A. geothermalis* ALDH recombinant plasmid was grown in LB medium containing the final concentration of 33 µg/mL chloramphenicol and 50 µg/mL ampicillin to an OD_600nm_~0.6. The recombinant cells were induced with Isopropyl β-d-1-thiogalactopyranoside (IPTG) at a final concentration of 0.75 mM. The enzyme was expressed at 25 °C for 20 h.

All steps in the following procedures were conducted at 4 °C. The *E. coli* cells were harvested by centrifugation at 10,000× *g* for 10 min at 4 °C and resuspended with binding buffer (20 mM Tris-HCL, 300 mM NaCl, 12 mM imidazole, pH 7.4). The cells were mixed thoroughly, then placed on ice for sonication using Branson sonifier 250 (output: 2, duty cycle: 30 and 3 min) and cleared by centrifugation (18,000× *g* for 10 min at 4 °C). The purification was done using AKTA Purifier. The clear crude lysate was applied to a Ni-Sepharose FAST Flow affinity chromatography. Unbound proteins were washed out with binding buffer and the target protein was eluted using elution buffer (20 mM Tris-HCl, 300 mM NaCl, 500 mM imidazole, pH 7.4). The eluted protein was then dialyzed against 20 mM Tris-HCl (pH 8.0). The purity and protein concentration were determined using the 12% SDS-PAGE (UVITec1d-quantification analysis) and Bradford method.

### 2.6. A. geothermalis ALDH Activity Assay

The assay was conducted using a method from Sigma Aldrich with a slight modification. The reaction mixture was composed of 50 mM Tris buffer (pH 8), 20 mM NADH, 50 mM of acetaldehyde, and 10 µL of the enzyme. The mixture without enzyme was used as a control. The assay was conducted at 60 °C for 10 min. The change in absorbance caused by the formation of NAD^+^ due to the oxidation of substrate by *A. geothermalis* ALDH was monitored at 340 nm using a spectrophotometer (Biotek Synergy 2, Santa Clara, CA, USA). One unit of *A. geothermalis* ALDH catalyzes the oxidation of 1.0 mmol reduced nicotinamide adenine dinucleotide, NADH, to the oxidized form, NAD per minute under assay conditions.

### 2.7. Characterization of the Purified A. geothermalis ALDH

#### 2.7.1. Determination of Optimum Temperature and Temperature Stability for *A. geothemalis* ALDH

To determine the optimum temperature for *A. geothermalis* ALDH activity, the reaction mixture was incubated at different assay temperatures ranging from 30 to 90 °C for 10 min. For temperature stability, the mixture of buffer and enzymes was pretreated for 30 min [14] at different temperatures ranging from 30 to 90 °C and assayed at optimum temperature. The OD_340nm_ was recorded. The average (mean) results for *A. geothermalis* ALDH activity quantification were obtained after each experiment was repeated three times.

#### 2.7.2. Determination of Optimum pH and pH Stability for *A. geothermalis* ALDH

To determine the optimum pH for *A. geothermalis* ALDH activity, buffers at different pH values ranging from pH 4.0 to 12.0 were used in the reaction mixture individually. The assay was conducted at 60 °C for 10 min. The buffer system used included sodium acetate (pH 4.0–6.0), phosphate buffer (pH 7–9), Tris-HCl (pH 8.0–9.0), glycine-NaOH (pH 9.0–11.0), and sodium phosphate (pH 11.0–12.0). For pH stability, the mixture of buffer at different pH ranges and the enzyme was pretreated at 60 °C for 30 min and the enzyme assay was performed at 60 °C for 10 min. The average (mean) results for *A. geothermalis* ALDH activity quantification were obtained after each experiment was repeated three times.

#### 2.7.3. Determination of the Effects of Metal Ions on *A. geothermalis* ALDH Stability

To determine the effect of metal ions on *A. geothermalis* ALDH activity, the enzyme was pretreated with Li^+^ (Li_2_Co_3_), Na^+^ (NaCl), K^+^ (KCl), Ca^2+^ (CaCl_2_), Mg^2+^ (MgCl_2_), Mn^2+^ (MnCl_2_), Fe^3+^ (Fe_2_(SO)_4_, Ni^2+^ (NiSO_4_), and Cu^2+^ (CuSO_4_) at a final concentration of 1 mM and 5 mM individually, at 60 °C for 30 min. The enzyme assay was conducted at 60 °C for 10 min. The average (mean) results for *A. geothermalis* ALDH activity quantification were obtained after each experiment was repeated three times.

#### 2.7.4. Determination of the Effects of Organic Solvents on *A. geothermalis* ALDH Activity

To determine the effect of organic solvents on *A. geothermalis* ALDH activity, the enzyme was pretreated with 25% (*v*/*v*) of DMSO, methanol, 2-propanol, 1-propanol, benzene, toluene, xylene, octanol, n-hexane, n-heptane, and n-tetradecane at 60 °C for 30 min with shaking at 50 rpm. The enzyme assay was performed at 60 °C for 10 min. The average (mean) results for *A. geothermalis* ALDH activity quantification were obtained after each experiment was repeated three times.

#### 2.7.5. Determination of the Effects of Substrate on *A. geothermalis* ALDH Activity

The substrate specificity was determined using acetaldehyde (C2), benzaldehyde (C6), vanillin (C8), and retinaldehyde (C20). The enzyme assay was performed using 50 mM of acetaldehyde, benzaldehyde, vanillin, and retinaldehyde at 60 °C for 10 min. The retinaldehyde was dissolved in the methanol. The average (mean) results for *A. geothermalis* ALDH activity quantification were obtained after each experiment was repeated three times.

#### 2.7.6. Secondary Structure and Melting Point Estimation Using Circular Dichroism (CD)

The melting point of the purified *A. geothemalis* ALDH was determined using CD spectropolarimeter (JASCO J810-spectrapolarimeter, Tokyo, Japan) at a concentration of 1 mg/mL. The temperature was increased from 20 °C to 90 °C, with an increment of 1 °C per minute, and the absorbance signal was collected from wavelengths of 222 nm. For the secondary structure estimation, the reactions were carried out in 500 uL with 0.2 mg/mL enzyme and 5 mM Tris-HCl, pH 8. The temperature was set at 60 °C and the absorbance was read ranging from 260 to 190 nm.

## 3. Results 

### 3.1. Sequence Analysis of A. geothermalis ALDH

**Figure 1:**
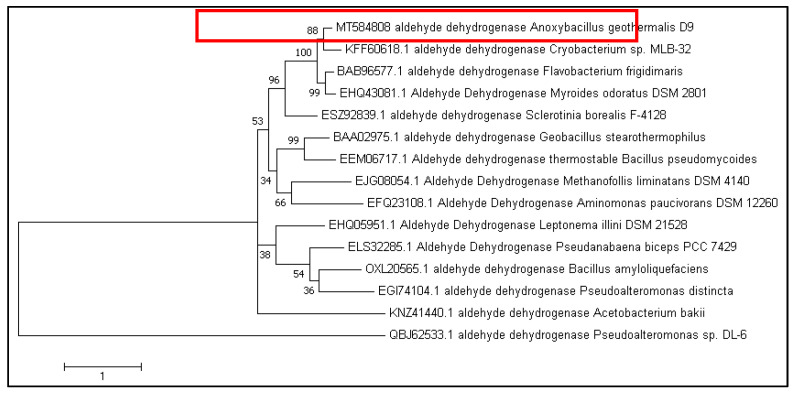
Phylogenetic tree of *A. geothermalis* ALDH from the *Anoxybacillus geothermalis* D9 (red box) and the related sequences of aldehyde dehydrogenases generated using MEGA 10.0. The amino acid sequences were retrieved from the National Center of Biotechnology (NCBI).

The *A. geothermalis* ALDH gene from newly isolated *Anoxybacillus geothermalis* D9 (MT584808) is coded by 1518 nucleotides. The predicted molecular weight and theoretical isoelectric point (pI) were 56 kDa and 5.54, respectively. The protein Blast using non-redundant protein sequence database at the National Center of Biotechnology (NCBI) (https://blast.ncbi.nlm.nih.gov/Blast.cgi, accessed on 16 March 2021) showed 99.8% amino acid sequence similarity of ALDH from *Anoxybacillus geothermalis* D9 with an unclassified *Anoxybacillus* strain. The constructed phylogenetic tree is based on the amino acid sequences of ALDH from *A. geothermalis* D9 and closely related to ALDH from other organisms (Figure 1). 

**Figure 2:**
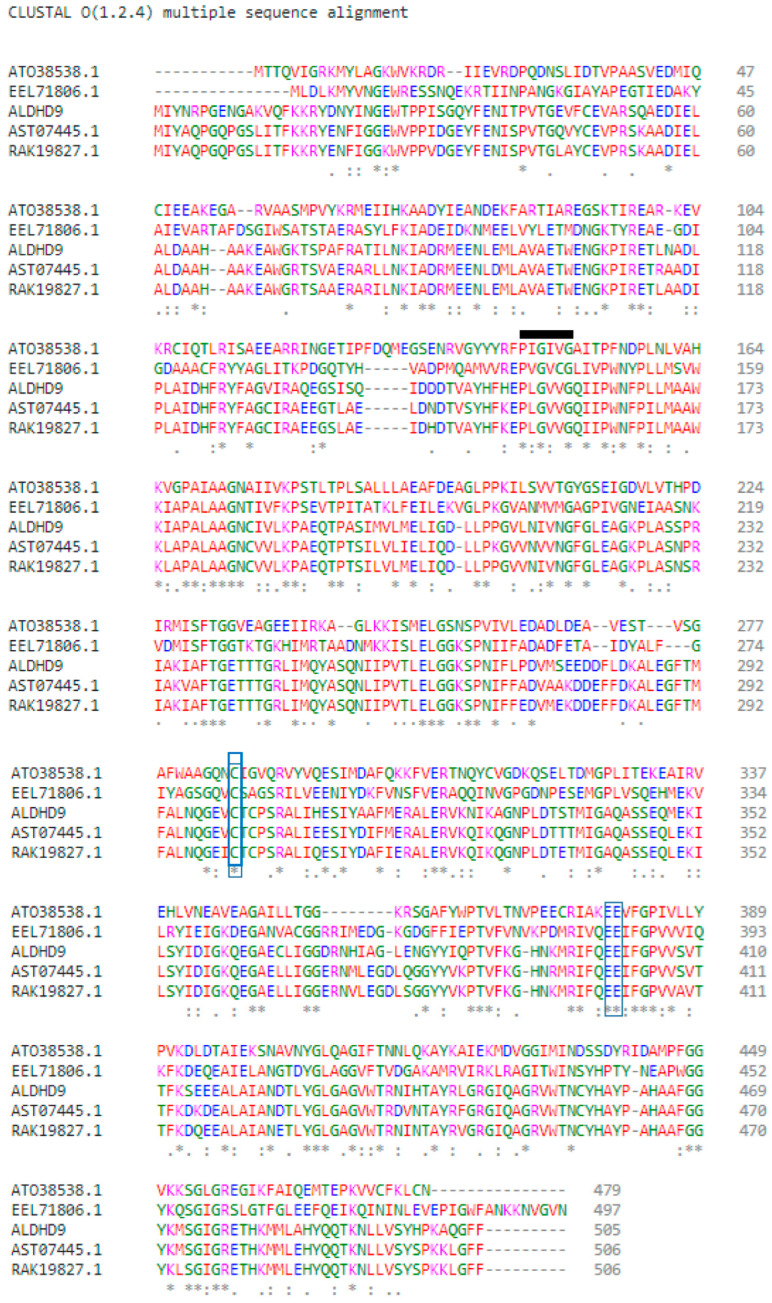
Multiple sequence alignment between the amino acid sequences of *A. geothermalis* ALDH from the *Anoxybacillus geothermalis* D9 (ALDH9) and Bacillaceae family. The * represents identical residue or strongly conserved region. The blue boxes show the catalytic residues, which are Cys301 and Glu399. The black line shows the motifs of *A. geothermalis* ALDH.

Multiple sequence alignment was used to identify the conserved regions, motifs, and active sites of the enzyme, based on the sequence comparison with other ALDH from Bacillaceae family such as *Anoxybacillus geothermalis* strain D9 (MT584808)*, Geobacillus thermodenitrificans* (AT038538.1), *Bacillus mycoides* (EEL71806.1), *Anoxybacillus flavithermus* (AST07445.1), and *Anoxybacillus vitaminiphilus* (RAK19827.1) (Figure 2). The monomer structure of *A. geothermalis* ALDH is composed of three domains common to all ALDHs, namely the NAD(P)^+^ binding domain, catalytic domain, and oligomerization domain.

The active site of the *A. geothermalis* ALDH comprises cysteine (Cys301) and glutamate (Glu399). Cysteine functions as a catalytic nucleophile, while glutamate activates a water molecule in the deacylation step [15]. The motif of the *A. geothermalis* ALDH is Gly-X-Gly-X-X-Gly (GXGXXG). For this *A. geothermalis* ALDH, the linear sequence closely resembles the motif, Pro-Leu-Gly-Val-Val-Gly, as depicted in the black line (Figure 2). 

### 3.2. Structure Prediction and Validation of A. geothermalis ALDH

Yet Another Scientific Artificial Reality Application was used to model *A. geothermalis* ALDH homology (YASARA). The software predicts the structure using one of two methods: comparative modelling or the de novo structure prediction method. The homosapiens aldehyde dehydrogenase crystal structure (1NZX A) was chosen as the template to build a 3D structure of *A. geothermalis* ALDH because it produced a higher score of sequence identity and the structure has already been solved using the X-ray diffraction method. We evaluated protein models with 3D profiles using the predicted structure of *A. geothermalis* ALDH as a topic (Table 1). VERIFY 3D showed 96.23 percent analyzed over the 0.2. (3D-1D). By contrasting the subject to previously solved structures, it was utilized to assess the accuracy of the 3D atomic model *A. geothermalis* ALDH received a high score of 95.18% on the Errat tool, which was used to evaluate the accuracy and correctness of the atom distribution in the protein area. A Ramachandran plot was used to verify the projected structure and showed that 90.4% of it, or 455 residues, were in the preferred region, with the remaining 9.1%, 0.3%, and 0.2% being located in the allowed, general, and disallowed regions, respectively. Overall, the predicted structure has been validated as a reliable structure that may be used for further research.

**Figure 3:**
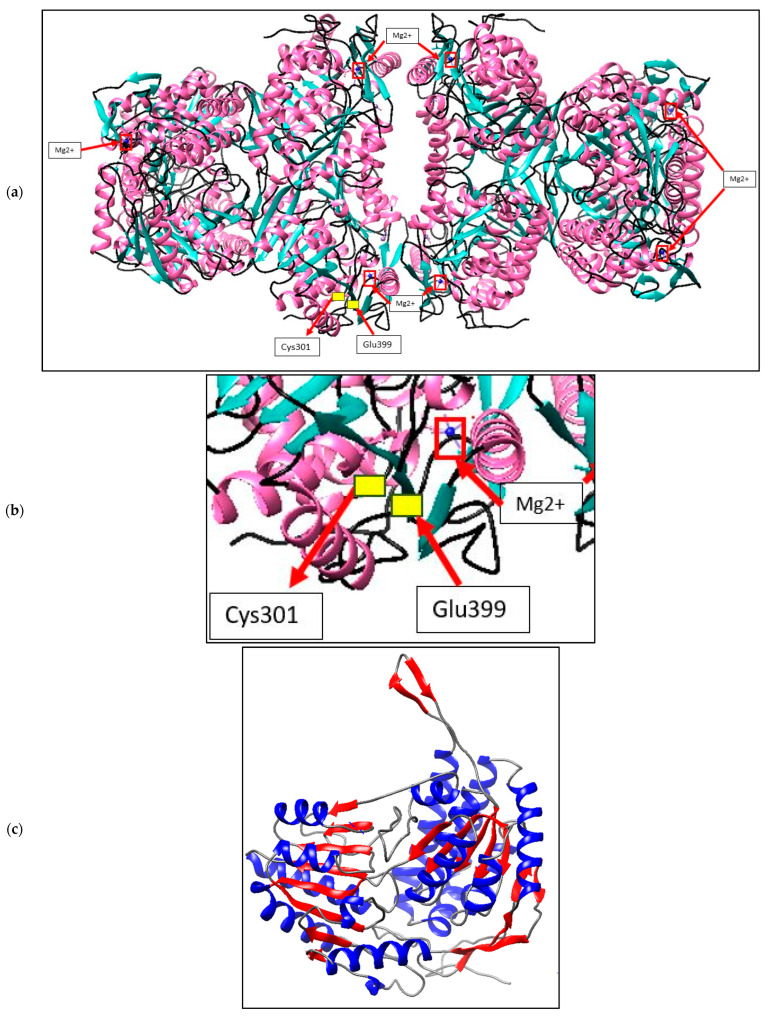
Structure prediction of *A. geothermalis* ALDH using YASARA software. (**a**) The predicted *A. geothermalis* ALDH exists as a tetramer composed of four monomers of the subunits. The red boxes represent the metal ions, Mg^2+^. The yellow boxes showed the active site of this *A. geothermalis* ALDH which are Cys301 and Glu399. (**b**) Active site and metal binding site of *A. geothermalis* ALDH (**c**) the monomer of the predicted *A. geothermalis* ALDH. The β-sheets coloured with red and the ɑ-helix coloured with blue.

ALDH enzymes are usually found as homotetramers or homodimers, with subunits ranging from 450 to 500 amino acids forming a 50–60 kDa protomer. Figure 3a depicts the expected *A. geothermalis* ALDH model, which is a tetramer made up of four monomers of the subunits. The ɑ-helix takes up 39.7% of each monomer, followed by 24.3% and 34.8% of β-sheets and others, respectively. Furthermore, the presence of a greater number of -sheets in the structure may aid the *A. geothermalis* ALDH’s thermostability. Figure 3b depicts an *A. geothermalis* ALDH monomer.

### 3.3. Expression and Purification of Recombinant A. geothermalis ALDH

The gene of *A. geothermalis* ALDH was cloned and expressed in an *E. coli* Transetta (DE3)/pET51b+ expression system. To increase the expression yield, routine optimization was carried out. Effects of temperature, induction time, and isopropyl β-D-1-thiogalactopyranoside (IPTG) concentration were investigated at Figures S1–S4 (data can be found at the supplemental results) and based on the optimization study, optimum expression of *A. geothemalis* ALDH was achieved with a final concentration of 0.75 mM IPTG induction at 25 °C for 20 h with the activity 350 U/mL. 

**Figure 4:**
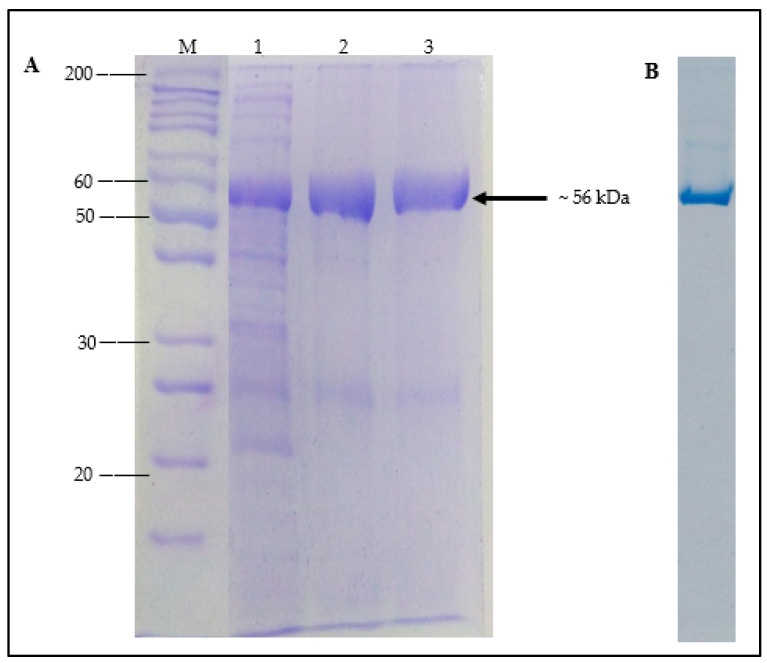
SDS-PAGE analysis of purified *A. geothermalis* ALDH. (**A**) SDS-PAGE analysis of *A. geothermalis* ALDH after affinity chromatography step using Ni-Sepharose Fast Flow. Lane M: Unstained protein molecular weight marker, Lane 1: crude cell lysate, Lane 2: pooled purified, Lane 3: dialysed purified *A. geothermalis* ALDH. (**B**) Native PAGE of purified *A. geothermalis* ALDH.

The soluble fraction of *A. geothermalis* ALDH was purified using single Ni-Sepharose affinity chromatography. The poly-histidine (His-tag) in the pET51b(+) plasmid was fused to the C-terminal of *A. geothermalis* ALDH to accelerate and ease the purification of the target protein by affinity chromatography [16]. The bound protein was eluted using an increasing imidazole concentration gradient after crude *A. geothermalis* ALDH was loaded into a Ni-Sepharose column. The bound protein was eluted at 500 mM concentration, and *A. geothermalis* ALDH activity assay and SDS-PAGE were used to determine the presence of the target protein. On SDS-PAGE, Figure 4A shows a dominant band with a molecular weight of ~56 kDa. From the quantification software (UniTec1d), the dominant band of *A. geothermalis* ALDH showed more than 90% purity. Native-PAGE was conducted to further determine the purity level of the *A. geothermalis* ALDH. As shown in Figure 4B, the *A. geothermalis* ALDH was purified to homogeneity, as only a single band was observed on the gel. 

In one-step purification of *A. geothermalis* ALDH, a 2.83-fold purification was achieved with a final yield of 37.98% (Table 2), indicating the successful purification of *A. geothermalis* ALDH. 

### 3.4. Characterization of Purified A. geothermalis ALDH

#### 3.4.1. Effect of Temperature on *A. geothermalis* ALDH Activity and Stability

**Figure 5:**
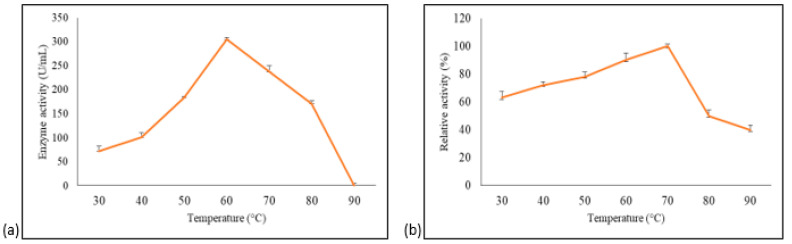
Temperature optimum and stability of the purified *A. geothermalis* ALDH. (**a**) Effect of temperature on the activity of aldehyde dehydrogenase. (**b**) Effect of temperature on the stability of aldehyde dehydrogenase.

The effect of temperature and stability on purified *A. geothermalis* ALDH was measured using acetaldehydehyde as a substrate. The impact of temperature on enzyme was assayed at different temperatures, ranging from 30 °C to 90 °C, with 10 °C intervals. The optimum temperature for *A. geothermalis* ALDH activity was 60 °C with activity at 288 U/mL, and the activity gradually dropped at temperature above 70 °C, as shown in Figure 5a. 

The stability study was attempted by pre-incubating the enzyme for 30 min at different temperatures ranging from 30 °C to 90 °C with 10 °C intervals before being assayed at 60 °C [14]. The results showed that aldehyde dehydrogenase is most stable at 70 °C, as shown in Figure 5b. The enzyme activity at 30 °C pre-incubation was approximately 40% lower compared to 70 °C. The activity gradually increased up to 100% when observed at 70 °C. A drastic decline in activity at temperature above 70 °C was probably due to the disruption of hydrogen bonds that are affected by prolonged thermal exposure [14]. 

#### 3.4.2. Effect of pH on *A. geothermalis* ALDH Activity and Stability

**Figure 6:**
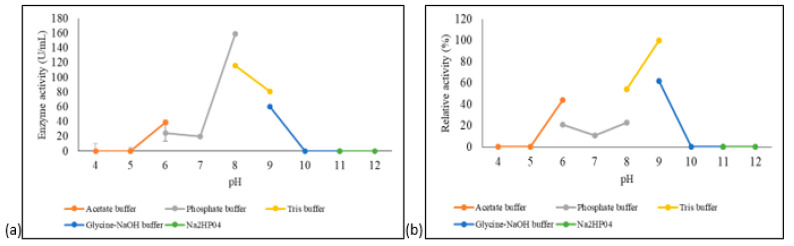
pH optimum and stability of the purified *A. geothermalis* ALDH. (**a**) Effect of pH on the activity of aldehyde dehydrogenase. (**b**) Effect of pH on the stability of aldehyde dehydrogenase.

The effects of pH on the activity and stability of *A. geothermalis* ALDH were tested using different buffer systems at pH ranging from 4–12. As shown in Figure 6a, the optimum pH for *A. geothermalis* ALDH was at pH 8 in phosphate buffer, while the activity dropped at acidic and highly alkaline pH.

The pH stability profile of *A. geothermalis* ALDH is shown in Figure 6b. *A. geothermalis* ALDH is most stable in Tris-HCl pH 9 compared to Tris-HCl, pH 8. The activity of *A. geothermalis* ALDH was reduced to less than 50% at pH 6–8. 

#### 3.4.3. Effects of Metal Ions on *A. geothermalis* ALDH Stability

As shown in Table 3, Mg^2+^ and Li^+^ at a final concentration of 1 mM enhanced the activity of *A. geothermalis* ALDH, which was higher than the control (enzyme without metal ions). Mg^2+^ at a final concentration of 5 mM retained 100% activity of *A. geothermalis* ALDH whileLi^+^, Cu2^+^, Fe^3+^, Mn^2+^, and Ni^2+^ ions at 5 mM completely inhibited the activity of *A. geothermalis* ALDH. 

#### 3.4.4. Effects of Organic Solvents on *A. geothermalis* ALDH Activity

As shown in Table 4, purified *A. geothermalis* ALDH treated with 25% and 30% (*v*/*v*) of various organic solvents are stable in most of the organic solvents used, except for toluene. With a low log *p*-value, purified *A. geothermalis* ALDH was stable in water-miscible solvents. In the presence of 1-propanol and benzene, high stability was observed, with a 60% and 75% increase in their activity compared to the control (without solvent). Similar results were reported by the thermoalkalophilic lipases from *Bacillus thermocatenulatus* where the activity of the lipases enhanced with the presence of polar solvents, 2-propanol [15]. In the presence of 25% of DMSO, methanol, xylene, n-hexane, octanol, 2-propanol, n-heptane and n-tetradecane, the activity of the *A. geothermalis* ALDH remained stable, as it can maintain an activity level of more than 50%. In the presence of 30% organic solvents, the activity of the *A. geothermalis* ALDH was completely inhibited (data not shown). 

#### 3.4.5. Substrate Specificity of Purified *A. geothermalis* ALDH

**Figure 7:**
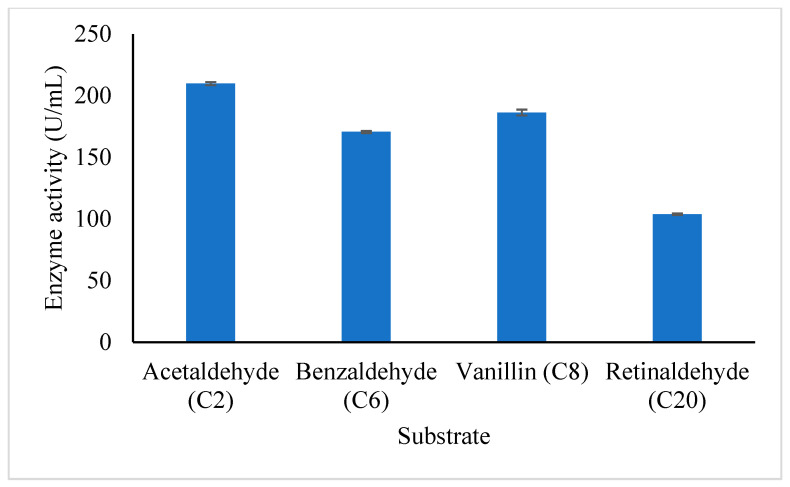
Effect of aliphatic and aromatic substrate on the purified *A. geothermalis* ALDH. The *A. geothermalis* ALDH was assayed at 60 °C for 10 min.

The study of the substrate specificity of *A. geothermalis* ALDH was conducted on both aliphatic and aromatic substrates which are acetaldehyde (C2), benzaldehyde (C6), vanillin (C8), and retinaldehyde (C20) as shown in Figure 7. The *A. geothemalis* ALDH showed high substrate specificity toward acetaldehyde (C2) as compared to the other substrates. The lowest activity of the *A. geothermalis* ALDH was observed when the *A. geothermalis* ALDH was assayed using retinaldehyde. A similar result was reported by [16], in which the activity of human liver aldehyde dehydrogenase (ALDH2) showed the highest activity using the acetaldehyde as a substrate. In contrast, no activity was observed when the ALDH2 was assayed using retinaldehyde. 

#### 3.4.6. Thermal Denaturation and Secondary Structure Analysis of Purified *A. geothermalis* ALDH

Circular dichroism (CD) is being increasingly recognized as a valuable technique for examining the structure of protein in solution. CD has been used to measure secondary structure estimation and thermal denaturation of the *A. geothermalis* ALDH. 

**Figure 8:**
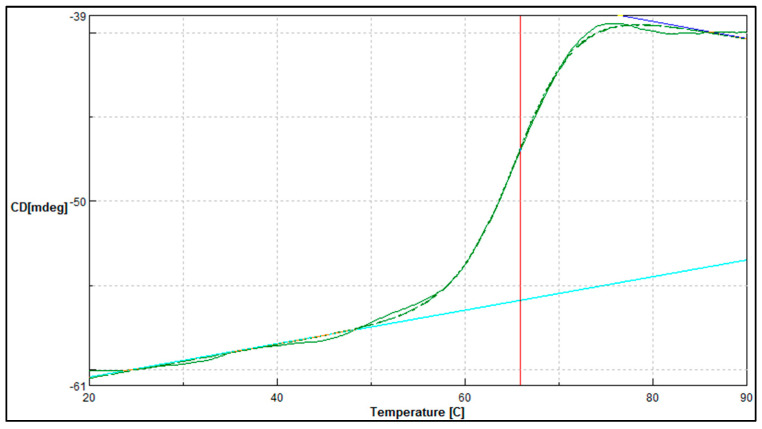
Thermal denaturation profile of purified *A. geothemalis* ALDH. Vertical line (red) indicates the melting point value when tested from temperature ranging from 20 to 90 °C.

In complement to the temperature stability study, the midpoint between the folded and unfolded state of the *A. geothermalis* ALDH was determined using JASCO J810-spectrapolarimeter (Figure 8). The measurement was conducted at 222 nm for temperatures ranging from 20 to 90 °C. The melting point for the *A. geothermalis* ALDH is 65.9 °C is in correlation with the temperature stability result that previously showed an activity drop after the enzyme was incubated at a temperature of more than 70 °C. 

For secondary structure estimation, the CD spectra of purified *A. geothermalis* ALDH was analyzed between 190 nm to 260 nm at 60 °C to monitor the transition of α-helix and β-strand structures. Table 5 shows the pattern of the secondary structure estimation. The overall helical and strand content are often linked together to help stabilize the protein structure. From the table, the percentage of the α-helix took most of the *A. geothermalis* ALDH structure, which is 39.3%, and the result correlated with the predicted structure using YASARA. The percentage of the turn and coil are 19.1% and 20.6%, respectively. 

## 4. Discussion

The *A. geothermalis* ALDH gene from newly isolated *A. geothermalis* D9 (MT584808) is coded by 1518 nucleotides. The homology modelling of *A. geothermalis* ALDH was done using YASARA. The overall structure consists of three distinct conserved domains, the NAD(P) binding domain, the catalytic domain, and the oligomerization domain [17]. Based on the predicted structure, the location of β-sheets is at the center core region of the structure and holds the structure to make it more stable at high temperature. These features might help the *A. geothermalis* ALDH to withstand high temperatures. The β-sheets help the *A. geothermalis* ALDH to withstand the high temperature by hydrophobic contacts and backbone hydrogen bonding [18]. Other than that, the higher number of ɑ-helix in the structure also plays an important role in temperature tolerance. The hydrogen bonding keeps the enzyme rigid and help the enzyme to tolerate the high temperature as more energy needed to break the hydrogen bond [19]. A similar result was shown by the structure of thermophilic β-galactosidases which also has a higher number of ɑ-helix in its structure. Each enzyme subunit contains two metal ions, Mg^2+^ (red boxes in Figure 3a). These Mg^2+^ help in enhancement of the enzyme activity, since this *A. geothermalis* ALDH is the metal dependant enzyme [20]. Mg^2+^ can increase the activity of the enzyme sharply, which could be due to the demand of the enzyme to this metal in the structure [20].

The gene of *A. geothermalis* ALDH was cloned and expressed in an *E. coli* Transetta (DE3)/pET51b+ expression system. A protein band corresponding to *A. geothermalis* ALDH with an expected size of ~56 kDa was obtained, as visualized by sodium dodecyl sulphate-polyacrylamide gel electrophoresis (SDS-PAGE). The same results were reported by Zhao et al. [21], who estimated the size of acetaldehyde dehydrogenase from *Acinetobacter* sp. strain HBS-2 was ~57 kDa, which was cloned into the pET28a(+) expression vector and expressed in *E. coli* BL21. The other reported aldehyde dehydrogenase from *Sulfolobus tokodaii* strain 7 had a smaller size, which was ~50 kDa. This enzyme used pET15b as an expression vector [10]. The DEAE–cellulose column was also used to purify the aldehyde dehydrogenase isolated from human salivary in a single step [16]. Unlike AldB, which was isolated from *Escherichia coli* DH5, the enzyme was purified in five steps using DEAE-Sephacel, HAP affinity column, 20–60% (NH_4_)_2_SO_4_ pellet, Q-Sepharose, and 5′ AMP-Sepharose. This occurred because AldB failed to bind to the HAP affinity column [8].

Temperatures have several effects on enzymatic reactions. Temperature can affect the kinetic energy of the molecules, which will affect the frequency of collisions between substrate and enzyme as well as the energy of activation for the reaction [22]. A higher temperature than the optimum could cause irreversible conformational changes, leading to unfolding or aggregation of the enzyme which affects the shape of the active site, and this would result in reducing the reaction rate [23]. The optimum temperature for *A. geothermalis* ALDH activity was 60 °C with activity at 288 U/mL, and the activity gradually dropped over 70 °C. 

pH is an important factor that regulates the activities of most enzymes. The ionic bonds in the enzyme structure may be altered if the pH is changed. These bonds are critical for structural stability and help to determine the protein 3D characteristic and functioning [24]. The effect of pH on the activity and stability of *A. geothermalis* ALDH was tested using different buffer systems at pH ranging from 4–12. *A. geothermalis* ALDH was most active at pH 8. The same result was reported by Liu et al. [10], where the activity of ALDH from *Sulfolobus tokodaii* strain 7 was the highest when it was incubated with Tris-HCl, pH 8. The graph trend shows that the activity of the *A. geothermalis* ALDH started to increase at pH 7 and gradually decreased in Glycine-NaOH buffer. This is due to the isoelectric point (pI) of the *A. geothermalis* ALDH, which is 5.54. Furthermore, due to increased electrostatic repulsions, protein solubility rises when pH values move away from its pI. As a result, the *A. geothermalis* ALDH enzyme is less active at pH levels close to or over its pI, as the enzyme may be inactivated or aggregate during the therapy [25]. 

Metal ions are essential for the biological functions of enzymes, such as electron donors or acceptors, Lewis acids, or structural regulators [25]. The conformational changes in the enzyme from an inactive to an active form may be the cause of the increased enzyme activity in the presence of Mg^2+^ ion. It has been suggested that when the cofactor has been reduced, the pace of transition from the closed to the open conformation is accelerated by the strong binding of the Mg^2+^ ion to NADH. The activity of ALDH from *Acinetobacter* sp. strain HBS-2 is also enhanced by the presence of Mg^2+^. Similarly, the activity of ALDH from *Escherichia coli DH5α* (aldB) increased with the presence of Mg^2+^ [8]. The substantial suppression of *A. geothermalis* ALDH activity by Li^+^, Cu2^+^, Fe^3+^, Mn^2+^, and Ni^2+^ ions could be due to their strong affinity for sulfhydryl groups and the formation of complexes with sulfhydryl groups on the side chains of specific amino acids. These findings suggested that *A. geothermalis* ALDH requires free sulfhydryl groups for the enzymatic activity or protein conformational integrity [26].

The partition coefficient, often known as *log p*, is a metric for the hydrophobicity of organic solvents. In hydrophilic (water miscible) solvents, *log p* values are less than 1.0, while in hydrophobic (water immiscible) solvents, *log p* values are greater than 4.0. The *log p* value increases with the hydrophobicity of the solvent [27]. According to studies, hydrophobic solvents have higher enzyme activity than hydrophilic solvents [27]. When biocatalysis takes place in organic solvents media, the *log p* value of the organic solvents has an impact on enzyme flexibility. The *A. geothermalis* ALDH resistance to denaturation by organic solvents was indicated by its stability in an aqueous–organic combination. One explanation is that *A. geothermalis* ALDH outer half is more hydrophilic and hence binds to water more tightly, allowing the biocatalyst to function in organic solvents [27]. Furthermore, the activity of *A. geothermalis* ALDH was completely lost with the presence of toluene. As reported by Shehata et al., [27], the activity of the lipases decreased due to the high stiffness in toluene. The decrease in the *A. geothermalis* ALDH activity may be related to this case.

The thermal denaturation study was conducted using JASCO J810-spectrapolarimeter in complement to the temperature stability study. This is an excellent tool to measure the change of state or thermodynamic unfolding of the polypeptide when applied as a function of temperature. When the heat was applied to the protein, the folded protein structure began to deteriorate until it reached its melting temperature [28]. As shown in Figure 8, the melting temperature for the purified *A. geothermalis* ALDH was 65.87 °C. The reduction of the activity may be caused by the disruption of the active site of the protein [29]. This might be due to irreversible conformational changes leading to unfolding, aggregation, and a decrease in catalytic efficacy.

Circular dichroism (CD) is becoming more widely acknowledged as a useful tool for studying protein structure in solution. Since CD signals only appear where light is absorbed, spectral bands may be easily ascribed to different structural characteristics of a molecule [28]. βALDH’s overall structure consisted primarily of ɑ-helix and β-sheet, which housed the catalytic region. The *A. geothermalis* ALDH stability and activity were improved by the high percentage of ɑ-helix and β-sheet [30]. The structure of the purified *A. geothemalis* ALDH is stable at 60 °C, and this is related to its temperature stability whereby the purified *A. geothermalis* ALDH is stable between 50 and 70 °C. 

## 5. Conclusions

A thermostable and organic solvent-tolerant *A. geothermalis* ALDH was isolated from the newly isolated *Anoxybacillus geothermalis* strain D9. The predicted structure of *A. geothermalis* ALDH revealed that it exists as a tetramer comprised of four monomers of the subunits. The overall structure of the *A. geothermalis* ALDH consists of three distinct conserved domains: the NAD(P) binding domain, the catalytic domain, and the oligomerization domain. Biochemical characterization revealed that it is stable at high temperatures and at pH 8–9. Studied against metal ions showed *A. geothermalis* ALDH was activated in the presence of Mg^2+^ and Li^+^. Additionally, this enzyme was found to be an organic-solvent-tolerant enzyme, the application of which is suited for industrial purposes. Biophysical characterization of this enzyme provides greater evidence that this enzyme can withstand high temperatures. The understanding of the characteristics of this thermostable *A. geothermalis* ALDH from *A. geothermalis* strain D9 can provide useful information for application in industry. 

## Figures and Tables

**Table 1:** The summary score for the predicted structure of *A. geothemalis* ALDH using online web tools.

Validation Tools		Score (%)
A	Verify 3D	96.23
B	Errat	95.18
C	Ramacandran plot	
	Most favoured region	90.4
	Additional allowed region	9.1
	Generously allowed region	0.3
	Disallowed region	0.2

**Table 2:** Purification table of purified *A. geothemalis* ALDH using nickel sepharose affinity chromatography.

Purification Step	Fraction Volume (mL)	Protein Content (mg/mL)	Protein Activity (U/mL)	Total Protein (mg)	Total Activity (U)	Specific Activity (U/mg)	Yield (%)	Fold-Purification
Crude extract	15	10.53	717.65	157.95	7764.75	49.18	100	1
Affinity chromatography	8	2.65	368.63	21.2	2949.04	139.11	37.98	2.83

**Table 3:** Effect of various metal ions on the stability of purified *A. geothermalis* ALDH.

Metal Ions/Inhibitors	Concentration (mM)	Relative Activity (%) ± SE
**Control**	-	100
**Li^+^**	1	138.83 ± 0.4
	5	34.95 ± 0.9
**Na^+^**	1	74.55 ± 0.3
	5	65.05 ± 0.6
**K^+^**	1	77.67 ± 0.2
	5	63.11 ± 0.6
**Ca^2+^**	1	97.09 ± 0.2
	5	72.82 ± 0.3
**Mg^2+^**	1	205.83 ± 0.8
	5	100.98 ± 0.9
**Mn^2+^**	1	69.9 ± 0.6
	5	45.63 ± 0.4
**Fe^3+^**	1	6.3 ± 0.1
	5	4.1 ± 0.5
**Ni^2+^**	1	63.11 ± 0.5
	5	49.69 ± 0.9
**Cu^2+^**	1	65.04 ± 0.7
	5	44.79 ± 0.8

Note: Values are means of three replicates ± SE.

**Table 4:** Stability of purified *A. geothermalis* ALDH in the presence of various organic solvents.

Solvents	Log *p*	Relative Activity (%) ± SE
**Control**	-	100
**DMSO**	−1.3	55.21 ± 1.8
**Methanol**	−0.76	65. 41 ± 2.7
**1-Propanol**	0.28	160.26 ± 3.2
**2-Propanol**	1.2	102.00 ± 0.6
**Benzene**	2	175.11 ± 2.8
**Toluene**	2.5	25.11 ± 1.2
**Octanol**	3	82.43 ± 2.3
**Xylene**	3.1	52.66 ± 3.9
**n-Hexane**	3.9	55.97 ± 2.8
**n-Heptane**	4.66	101.26 ± 1.4
**n-Tetradecane**	7.2	64.47 ± 0.9

Note: Values are means of three replicates ± SE.

**Table 5:** Secondary structure determination of *A. geothermalis* ALDH.

Secondary Structure	Amount of Secondary Structure (%)
α-helix	39.3
β-sheet	21.6
Turn	19.1
Coil	20.6

## Data Availability

All data generated or analyzed during this study are included in this published article.

## Supplementary Materials

The following supporting information can be downloaded at: https://www.mdpi.com/article/10.3390/microorganisms10071444/s1, Figure S1: Effect of expression hosts on the expression of aldehyde dehydrogenase; Figure S2: Effect of induction time on the expression of aldehyde dehydrogenase; Figure S3: Effect of temperature on the expression of aldehyde hydrogenase; Figure S4: Effect of concentration of inducer on the expression of aldehyde dehydrogenase; Figure S5: modelling of ALDH by using the SWISS MODEL; Figure S6: Homology modelling of ALDH by using the RaptorX; Figure S7: Chromatogram profile of the purified ALDH.

## References

[1] Robinson P.K. (2015). Enzymes: Principles and biotechnological applications. Essays Biochem..

[2] Haki G.D., Rakshit S.K. (2003). Developments in industrially important thermostable enzymes: A review. Bioresour. Technol..

[3] Kumar S., Tsai C.J., Nussinov R. (2000). Factors enhancing protein thermostability. Protein Eng..

[4] Hayes K., Noor M., Djeghader A., Armshaw P., Pembroke T., Tofail S., Soulimane T. (2018). The quaternary structure of Thermus thermophilus aldehyde dehydrogenase is stabilized by an evolutionary distinct C-terminal arm extension. Sci. Rep..

[5] Li X., Li Y., Wei D., Li P., Wang L., Feng L. (2010). Characterization of a broad-range aldehyde dehydrogenase involved in alkane degradation in *Geobacillus thermodenitrificans* NG80-2. Microbiol. Res..

[6] Perozich J., Nicholas H., Wang B.C., Lindahl R., Hempel J. (1999). Relationships within the aldehyde dehydrogenase extended family. Protein Sci..

[7] Stagos D., Chen Y., Brocker C., Donald E., Jackson B.C., Orlicky D.J., Thompson D.C., Vasiliou V. (2010). Aldehyde dehydrogenase 1B1: Molecular cloning and characterization of a novel mitochondrial acetaldehyde-metabolizing enzyme. Drug Metab. Dispos..

[8] Ho K.K., Weiner H. (2005). Isolation and characterization of an aldehyde dehydrogenase encoded by the aldB gene of *Escherichia coli*. J. Bacteriol..

[9] Kim H.J., Joo W.A., Cho C.W., Kim C.W. (2006). Halophile Aldehyde Dehydrogenase from *Halobacterium salinarium*. J. Proteome Res..

[10] Liu T., Hao L., Wang R., Liu B. (2013). Molecular characterization of a thermostable aldehyde dehydrogenase (ALDH) from the hyperthermophilic archaeon *Sulfolobus tokodaii* strain 7. Extremophiles.

[11] Yusoff D.F., Raja Abd Rahman R.N., Masomian M., Ali M.S., Leow T.C. (2020). Newly isolated alkane hydroxylase and lipase producing *geobacillus* and *anoxybacillus* species involved in crude oil degradation. Catalysts.

[12] Bankevich A., Nurk S., Antipov D., Gurevich A.A., Dvorkin M., Kulikov A.S., Lesin V.M., Nikolenko S.I., Pham S., Prjibelski A.D. (2012). SPAdes: A new genome assembly algorithm and its applications to single-cell sequencing. J. Comput. Biol..

[13] Overbeek R., Olson R., Pusch G.D., Olsen G.J., Davis J.J., Disz T., Edwards R.A., Gerdes S., Parrello B., Shukla M. (2014). The SEED and the Rapid Annotation of microbial genomes using Subsystems Technology (RAST). Nucleic Acids Res..

[14] Yamanaka Y., Kazuoka T., Yoshida M., Yamanaka K., Oikawa T., Soda K. (2002). Thermostable aldehyde dehydrogenase from psychrophile, *Cytophaga* sp. KUC-1: Enzymological characteristics and functional properties. Biochem. Biophys. Res. Commun..

[15] Tsybovsky Y., Krupenko S.A. (2011). Conserved catalytic residues of the ALDH1L1 aldehyde dehydrogenase domain control binding and discharging of the coenzyme. J. Biol. Chem..

[16] Maejima R., Iijima K., Kaihovaara P., Hatta W., Koike T., Imatani A., Salaspuro M. (2015). Effects of ALDH2 genotype, PPI treatment and L-cysteine on carcinogenic acetaldehyde in gastric juice and saliva after intragastric alcohol administration. PLoS ONE.

[17] Shortall K., Djeghader A., Magner E., Soulimane T. (2021). Insights into aldehyde dehydrogenase enzymes: A structural perspective. Front. Mol. Biosci..

[18] Numata K., Cebe P., Kaplan D.L. (2010). Mechanism of enzymatic degradation of beta-sheet crystals. Biomaterials.

[19] Daniel R.M., Dines M., Petach H.H. (1996). The denaturation and degradation of stable enzymes at high temperatures. Biochem. J..

[20] Meaden P.G., Dickinson F.M., Mifsud A., Tessier W., Westwater J., Bussey H., Midgley M. (1997). The ALD6 gene of Saccharomyces cerevisiae encodes a cytosolic, Mg2+-activated acetaldehyde dehydrogenase. Yeast.

[21] Zhao Y., Lei M., Wu Y., Wang C., Zhang Z., Deng F., Wang H. (2009). Molecular cloning and expression of the complete DNA sequence encoding NAD+-dependent acetaldehyde dehydrogenase from *Acinetobacter* sp. strain HBS-2. Ann. Microbiol..

[22] Karplus M., McCammon J.A., Peticolas W.L. (1981). The internal dynamics of globular protein. Crit. Rev. Biochem..

[23] Shehata M., Timucin E., Venturini A., Sezerman O.U. (2020). Understanding thermal and organic solvent stability of thermoalkalophilic lipases: Insights from computational predictions and experiments. J. Mol. Model..

[24] Mohamad N.R., Marzuki N.H.C., Buang N.A., Huyop F., Wahab R.A. (2015). An overview of technologies for immobilization of enzymes and surface analysis techniques for immobilized enzymes. Biotechnol. Biotechnol. Equip..

[25] Masomian M., Rahman R.N., Salleh A.B. (2018). A Novel Method of Affinity Tag Cleavage in the Purification of a Recombinant Thermostable Lipase from *Aneurinibacillus thermoaerophilus* Strain HZ. Catalysts.

[26] Ajsuvakova O.P., Tinkov A.A., Aschner M., Rocha J.B., Michalke B., Skalnaya M.G., Skalny A.V., Butnariu M., Dadar M., Sarac I. (2020). Sulfhydryl groups as targets of mercury toxicity. Coord. Chem. Rev..

[27] Wang S., Meng X., Zhou H., Liu Y., Secundo F., Liu Y. (2016). Enzyme stability and activity in non-aqueous reaction systems: A mini review. Catalysts.

[28] Corrêa D.H., Ramos C.H. (2009). The use of circular dichroism spectroscopy to study protein folding, form and function. Afr. J. Biochem. Res..

[29] Singh K., Shandilya M., Kundu S., Kayastha A.M. (2015). Heat, acid and chemically induced unfolding pathways, conformational stability and structure-function relationship in wheat α-amylase. PLoS ONE.

[30] Rogers D.M., Jasim S.B., Dyer N.T., Auvray F., Réfrégiers M., Hirst J.D. (2019). Electronic circular dichroism spectroscopy of proteins. Chem.

