# Supplementary material for: Structure Prediction and Characterization of Thermostable Aldehyde Dehydrogenase from Newly Isolated Anoxybacillus geothermalis Strain D9
Source: Microorganisms. 2022 Jul 18;10(7):1444. doi: 10.3390/microorganisms10071444 (PMC9322625; doi:10.3390/microorganisms10071444)
Supplement: Supplementary file 1 [file microorganisms-10-01444-s001.zip › microorganisms-1759223-supplementary.pdf]

Supplemental results

Optimization of ALDH expressions

Effect of hosts on ALDH expression

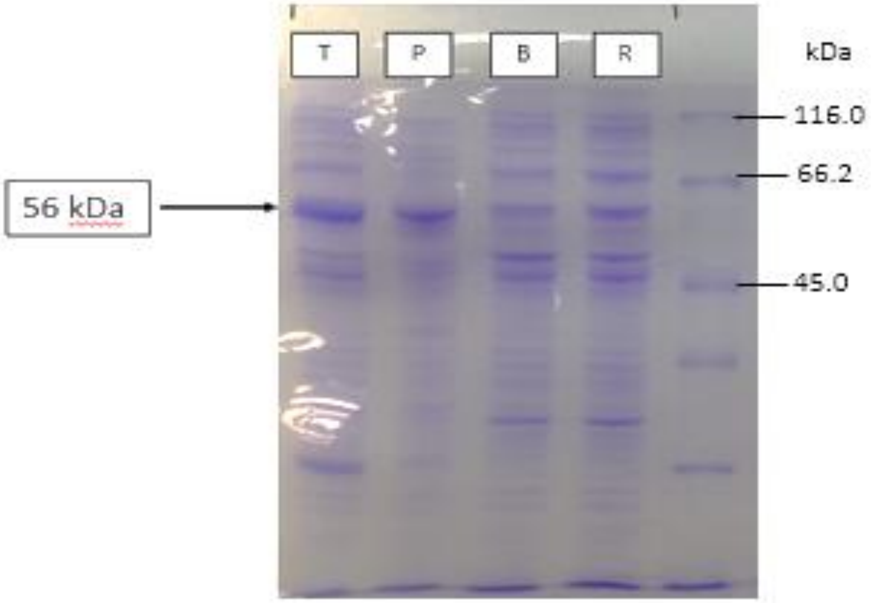

Figure S1: Effect of expression hosts on the expression of aldehyde dehydrogenase. M: Protein marker; T: *E. coli* Transetta (DE3); 2*: E. coli* Rosetta-gami (DE3); 3: *E. coli* BL21 (DE3) pLysS; 4*: E. coli* BL21 (DE3).

Effect of induction time on ALDH expression

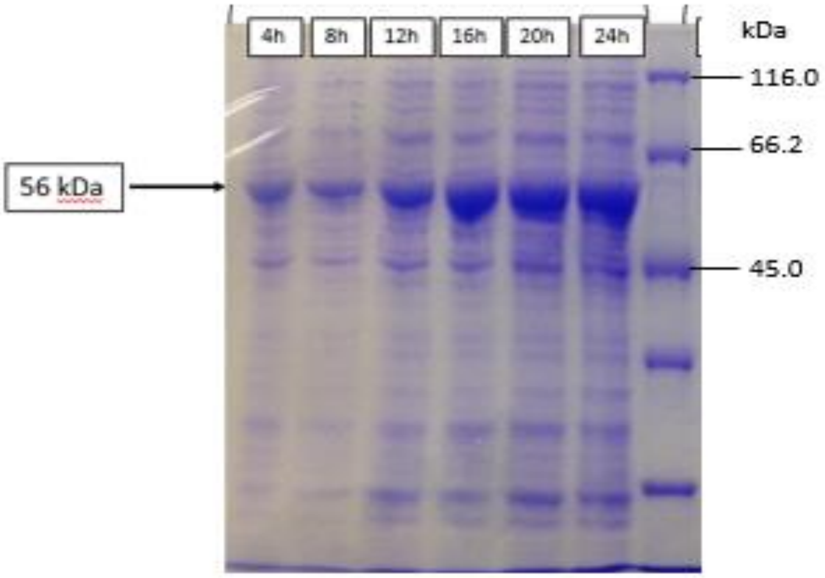

Figure S2: Effect of induction time on the expression of aldehyde dehydrogenase. M: Protein marker; 1: 4 h; 2: 8 h; 3: 12 h; 4: 16 h; 5: 20 h; 6: 24 h.

Effect of induction temperature on ALDH expressions

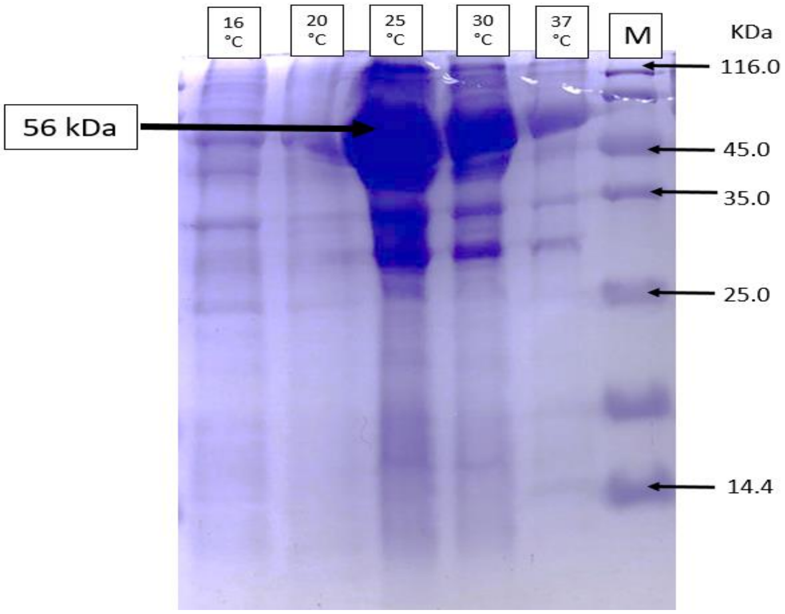

Figure S3: Effect of temperature on the expression of aldehyde hydrogenase. M: Protein marker; 1: 16 °C; 2: 20 °C; 3: 25 °C; 4: 30 °C; 5: 37 °C

Effect of IPTG concentration on ALDH expression

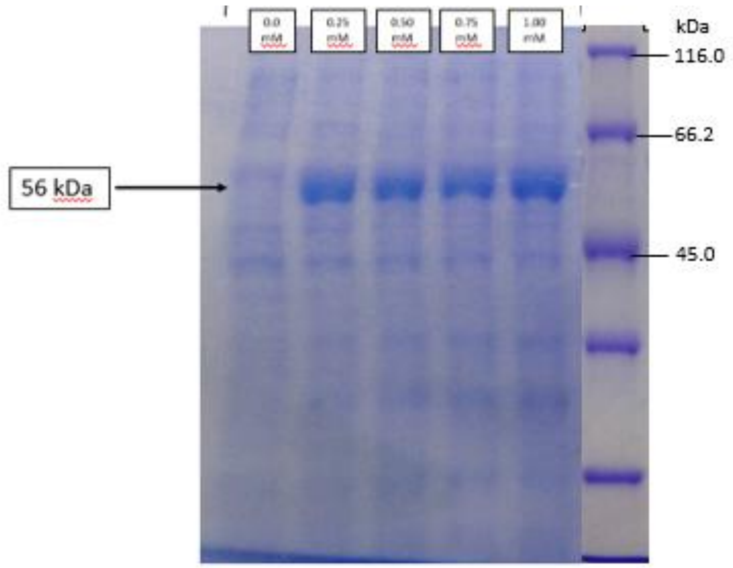

Figure S4: Effect of concentration of inducer on the expression of aldehyde dehydrogenase. M: Protein marker; 1: 0.00 mM; 2: 0.25 mM; 3: 0.50 mM; 4: 0.75 mM; 5: 1.00 mM

Homology modelling of ALDH

Enzymes	Program/ software	Template	Model	Validation					
				Verify 3D (%)	Errat (%)	Ramacandran plot			
						Favoured (%)	Allowed (%)	Generously allowed (%)	Disallowed (%)
Aldehyde dehydrogenase	SWISS MODEL	4O6R_A	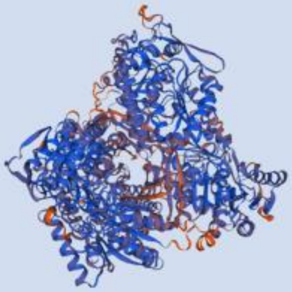	86.25	91.4405	90.4	8.8	0.5	0.4

Figure S5: Homology modelling of ALDH by using the SWISS MODEL.

Enzymes	Program/ software	Template	Model	Validation					
				Verify 3D (%)	Errat (%)	Ramacandran plot			
						Favoured (%)	Allowed (%)	Generousl y allowed (%)	Disallowe d (%)
Aldehyde dehydrogenase	RAPTORX	5FHZ_A	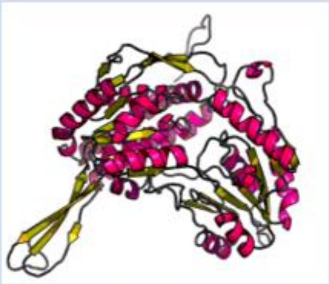	82.97	69.6099	91.5	6.5	1.2	0.5

Figure S6: Homology modelling of ALDH by using the RaptorX.

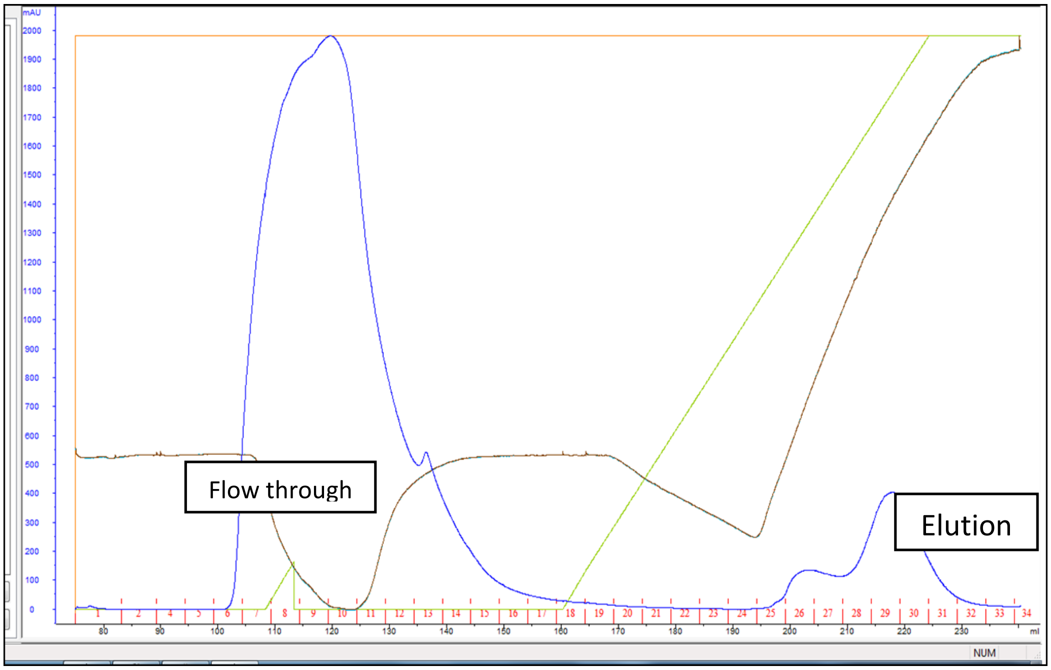

Figure S7: Chromatogram profile of the purified ALDH.
